# Topological Data Analysis of Ninjin'yoeito Effects Unraveling Complex Interconnections in Patients With Frailty: A Pilot Study

**DOI:** 10.7759/cureus.74855

**Published:** 2024-11-30

**Authors:** Nobuo Okui, Tadashi Ikegami, Machiko Okui

**Affiliations:** 1 Urogynecology, Yokosuka Urogynecology and Urology Clinic, Yokosuka, JPN; 2 Dentistry, Kanagawa Dental University, Yokosuka, JPN; 3 Diagnostic Imaging, Kanagawa Dental University, Yokosuka, JPN

**Keywords:** geriatrics, ninjin'yoeito, overactive bladder, personalized medicine, topological data analysis

## Abstract

Background

Ninjin'yoeito (NYT), a traditional Japanese Kampo medicine, has shown potential in treating frailty and overactive bladder (OAB) symptoms. However, its effects are multifaceted and vary among individuals. This pilot study explored the use of topological data analysis (TDA) and natural language processing (NLP) to evaluate the effect of NYT on frailty in patients with OAB.

Methods

Fifteen patients with frailty aged 75 or older underwent pelvic floor muscle training (PFMT) and one month of NYT administration. The eight standardized health questionnaires were simplified into a 28-item format using NLP. Persistent homology analysis via TDA revealed the complex, multidimensional effects of NYT, while network graph clustering using the Louvain method identified key health domains influenced by NYT.

Results

TDA revealed multiloop structures in the therapeutic effects of NYT, indicating multiple pathways of improvement across physical and mental health domains. Network graph clustering identified four distinct communities linking OAB symptoms with energy, physical function, mental stress, and sleep quality. No significant adverse effects were noted.

Conclusions

This pilot study demonstrated the feasibility of using TDA and NLP to analyze the complex effects of NYT on frailty in patients with OAB. These findings suggest that NYT exerts multifaceted therapeutic benefits and further large-scale studies are warranted to explore its long-term efficacy.

## Introduction

Ninjin'yoeito (NYT) is a traditional Japanese Kampo medicine composed of 12 crude drugs, and it has been proven effective in treating various symptoms in patients with frailty, especially those with overactive bladder (OAB) [1]. Previous studies have confirmed that this Kampo formulation is beneficial in improving genitourinary symptoms, enhancing hematopoietic function, and addressing frailty in patients with mild cognitive impairment and mild Alzheimer's disease [1-4]. Additionally, NYT has been shown to improve anemia, fatigue, and anxiety in patients with gynecological diseases [5], maintain weight and nutritional status in elderly patients with chronic wasting diseases [6], and enhance the quality of life after hospitalization for acute illnesses in patients with frailty [7]. However, significant individual variations in the effectiveness of NYT have been observed [1], and research using causal inference has clarified that while NYT is effective for OAB, its effects manifest differently across individuals [8]. 

Furthermore, it has been reported that while NYT can improve various symptoms of frailty, the specific symptoms it addresses vary from person to person [9]. A recent case report highlighted the diverse impact of NYT on symptoms such as fatigue, anxiety, and physical weakness in elderly women with frailty and OAB [9]. This underscores the individualized nature of NYT’s therapeutic effects, necessitating a deeper understanding of how it affects different patients across various health domains.

This variability necessitates a comprehensive evaluation of the effects of NYT, a complex Kampo formulation, on frailty in patients with OAB symptoms [8,9]. The primary objective of this study was to elucidate the relationship between NYT administration and various health outcomes in this patient population, focusing on understanding the interconnectedness of its effects. This study employed natural language processing (NLP) to condense assessments [10]. This approach resulted in a streamlined 28-item questionnaire that preserved comprehensive data collection while minimizing the burden on patients. Patients underwent one month of pelvic floor muscle training (PFMT), followed by one month of NYT administration, during which they completed the questionnaire and identified the three most significant effects they experienced. 

The Louvain method, which is typically used in social network analysis, was adopted to analyze the relationships between NYT effects [11,12]. This community detection algorithm efficiently identified clusters within networks, potentially revealing temporal changes in the impact of Kampo medicine. The application of this advanced network analysis technique to Kampo medical research represents a novel approach for understanding the complex, multifaceted effects of herbal medicines. 

By mapping the interconnected effects of NYT using topological data analysis (TDA), this study aimed to provide insights for personalized Kampo medicine, potentially leading to more targeted and effective treatments for patients with frailty and OAB symptoms. TDA, specifically persistent homology, allows for the identification of persistent patterns and structures within the data, capturing the complex, multifaceted nature of NYT effects across different patients [13]. This topological approach not only reveals the intricate connections among the effects of this Kampo formulation but also offers a novel framework for evaluating complex interventions in healthcare, paving the way for a deeper understanding of how traditional Japanese Kampo medicines exert their therapeutic benefits in diverse patient populations. 

## Materials and methods

Study design

This pilot study was approved by the Ethics Committee of Kanagawa Dental University and aimed to evaluate the efficacy of NYT, a traditional herbal medicine, in patients with frailty aged 75 years or older, using TDA for visualization. This is the first known application of TDA in clinical medicine. Fifteen patients were enrolled on a first-come, first-served basis from February 1, 2023. Data were collected at the Yokosuka Urogynecology and Urology Clinic, and statistical analyses were conducted at Kanagawa Dental University.

Eligibility criteria 

Participants were eligible if they were 75 years of age or older and met the criteria for frailty according to the Fried Frailty Criteria [14]. These criteria included unintentional weight loss, self-reported exhaustion, weakness (measured using grip strength), slow walking speed, and low physical activity. Frailty was defined as meeting three or more of these criteria, whereas pre-frailty was defined as meeting one or two. Additional exclusion criteria included the presence of acute diseases, such as infections or trauma, which could worsen the patient's condition in the short term. Patients with drug or alcohol dependence were excluded because these conditions could interfere with study adherence and outcomes. Moreover, the use of herbal medications was excluded to prevent potential interactions that could confound the results. Patients taking medications for OAB and those who had received botulinum toxin A injections into the bladder were also excluded from the study. Informed consent was obtained in written form from all participants.

Treatment schedule 

The treatment schedule involved several steps. First, all patients were registered and received instructions on PFMT [1], which they practiced for one month. After this initial period, patients completed a comprehensive 28-item questionnaire. They then began a one-month regimen of oral NYT administration in combination with continued pelvic floor muscle exercises. Subsequently, patients completed the same 28-item questionnaire again, allowing for a comparison of their health outcomes before and after treatment.

Pelvic floor muscle training

PFMT was administered by a physician and nurse at each appointment [1]. Patients were guided on how to contract their pelvic muscles using intravaginal digital palpation. An instructional video was shared on YouTube to assist patients in performing these exercises at home. They were instructed to practice PFMT for 30 minutes daily. During follow-up visits, the medical team reviewed the patient's PFMT exercise logs and provided encouragement to patients who adhered to the regimen.

NYT administration

The subjects received NYT granules (KB-108, Kracie Pharma Ltd., Tokyo, Japan) at a daily dose of 7.5 g. This medication was covered by insurance and dosed according to the recommendations of the Japanese Pharmaceuticals and Medical Devices Agency [1]. Medication adherence was monitored by a pharmacist who quantified any unused medications returned by the participants. If participants stopped taking the medication, they were considered to have participated for less than one month and were excluded from the study. In cases of adverse events, the NYT package information and medical assessments were used to determine any link between NYT use and adverse effects.

Questionnaire simplification

To reduce the complexity of data collection for patients with frailty, NLP techniques were applied to condense eight standardized health questionnaires into a more manageable 28-item format. The original questionnaires included the Short Form-36 (SF-36) (36 items) [15], Self-Rated Health (SRH) (five items) [9], Fried Frailty Criteria (five items) [14], Depression Anxiety Stress Scales-21 (DASS-21) (21 items) [16], Geriatric Depression Scale (GDS) (30 items) [17], Gastrointestinal Symptom Rating Scale (GSRS) (15 items) [18], Pittsburgh Sleep Quality Index (PSQI) (19 items) [9], and Overactive Bladder Symptom Score (OABSS) (four items) [1], for a total of 135 items. A preliminary test conducted on five patients with frailty revealed that, due to physical limitations, participants stopped answering after completing only approximately 50% of the items (data not shown). To address this issue and ensure that patients could complete the questionnaires while maintaining a fair comparison across health domains, particularly in relation to the shorter OABSS, we used to adjust and simplify the questionnaire format. The simplification process employed term frequency-inverse document frequency (TF-IDF) to rank the importance of terms in each questionnaire, followed by singular value decomposition (SVD) to reduce the dimensionality of the data [19]. The final result was a 28-item questionnaire that preserved the essential evaluative properties of each original instrument while minimizing the cognitive and physical burden on the patients. Table 1 lists the key questions from each domain after applying the NLP-based simplification process. Each domain was reduced to four representative questions while still capturing the critical aspects of patient health.

**Table 1:** Key questions for evaluating health domains in patients with frailty, simplified using an NLP-based approach. SF-36: Short Form-36; SRH: Self-Rated Health; DASS-21: Depression Anxiety Stress Scales-21; GDS: Geriatric Depression Scale; GSRS: Gastrointestinal Symptom Rating Scale; PSQI: Pittsburgh Sleep Quality Index; OABSS: Overactive Bladder Symptom Score; NLP: natural language processing.

Question No	Category	Questionnaire	Question	Answers
1	Energy and Vitality Improvement	SF-36, Fatigue Assessment Scale	How often do you feel fatigued in daily life?	1: Not at all 2: Sometimes 3: Often 4: Always
2	Energy and Vitality Improvement	SF-36, Fatigue Assessment Scale	Do you feel your vitality has increased recently?	1: Strongly agree 2: Somewhat agree 3: Disagree 4: Strongly disagree
3	Energy and Vitality Improvement	SF-36, Fatigue Assessment Scale	What is your energy level during physical activity?	1: High 2: Moderate 3: Low 4: Very low
4	Energy and Vitality Improvement	SF-36, Fatigue Assessment Scale	How long does it take for you to recover after activity?	1: Immediately 2: Takes some time 3: Takes a long time 4: Takes a very long time
5	Immune Function Improvement	Self-Rated Health Questionnaire	Do you feel you catch colds or infections less often recently?	1: Not at all 2: Significantly less 3: Slightly less 4: No change
6	Immune Function Improvement	Self-Rated Health Questionnaire	Do you feel your immune system has improved?	1: Strongly agree 2: Somewhat agree 3: Disagree 4: Strongly disagree
7	Immune Function Improvement	Self-Rated Health Questionnaire	How frequently have you been ill in the past 6 months?	1: Never 2: Rarely 3: Sometimes 4: Often
8	Immune Function Improvement	Self-Rated Health Questionnaire	How would you rate your overall health?	1: Excellent 2: Good 3: Fair 4: Poor
9	Muscle Strength and Physical Function Improvement	Frailty Evaluation Scale	How has your grip strength changed compared to before?	1: Improved significantly 2: Improved 3: No change 4: Weakened
10	Muscle Strength and Physical Function Improvement	Frailty Evaluation Scale	How do you feel about your muscle strength in daily activities (e.g., standing up, walking)?	1: Very strong 2: Strong 3: Weak 4: Very weak
11	Muscle Strength and Physical Function Improvement	Frailty Evaluation Scale	How much do you walk per day?	1: A lot 2: Moderate amount 3: A little 4: Almost none
12	Muscle Strength and Physical Function Improvement	Frailty Evaluation Scale	How is your stamina when climbing stairs?	1: Excellent 2: Good 3: Fair 4: Poor
13	Mental Stress Reduction	DASS-21, GDS	How often do you feel mental stress recently?	1: Not at all 2: Occasionally 3: Often 4: Very often
14	Mental Stress Reduction	DASS-21, GDS	Do you feel down or depressed?	1: Never 2: Rarely 3: Sometimes 4: Often
15	Mental Stress Reduction	DASS-21, GDS	How frequently do you feel anxious?	1: Not at all 2: Sometimes 3: Often 4: Always
16	Mental Stress Reduction	DASS-21, GDS	Do you feel joy in daily activities?	1: Always 2: Often 3: Sometimes 4: Never
17	Digestive Function Improvement	Gastrointestinal Symptom Rating Scale	How is your appetite compared to before?	1: Increased 2: No change 3: Decreased 4: Greatly decreased
18	Digestive Function Improvement	Gastrointestinal Symptom Rating Scale	How often do you experience indigestion (e.g., bloating, fullness)?	1: Never 2: Rarely 3: Sometimes 4: Often
19	Digestive Function Improvement	Gastrointestinal Symptom Rating Scale	Is your bowel movement regular?	1: Always 2: Often 3: Sometimes 4: Never
20	Digestive Function Improvement	Gastrointestinal Symptom Rating Scale	How often do you feel discomfort after eating?	1: Never 2: Rarely 3: Sometimes 4: Often
21	Sleep Quality Improvement	PSQI	How is the quality of your sleep recently?	1: Excellent 2: Good 3: Fair 4: Poor
22	Sleep Quality Improvement	PSQI	How often do you wake up at night?	1: Never 2: Rarely 3: Sometimes 4: Often
23	Sleep Quality Improvement	PSQI	How refreshed do you feel when you wake up in the morning?	1: Very refreshed 2: Refreshed 3: Somewhat tired 4: Very tired
24	Sleep Quality Improvement	PSQI	How easy is it for you to fall asleep?	1: Very easy 2: Easy 3: Difficult 4: Very difficult
25	Improvement of Overactive Bladder Symptoms	OABSS	How frequently do you urinate during the day?	1: Rarely 2: Occasionally 3: Often 4: Very often
26	Improvement of Overactive Bladder Symptoms	OABSS	How frequently do you wake up to urinate at night?	1: Never 2: Once 3: Twice 4: Three or more times
27	Improvement of Overactive Bladder Symptoms	OABSS	How often do you feel a sudden, urgent need to urinate?	1: Never 2: Rarely 3: Sometimes 4: Often
28	Improvement of Overactive Bladder Symptoms	OABSS	How often do you experience incontinence?	1: Never 2: Rarely 3: Sometimes 4: Often

Topological data analysis

TDA using persistent homology was applied to assess the interconnected effects of NYT across different health domains. This technique identifies "birth" and "death" of topological features, such as clusters and loops, within the data, allowing for the detection of persistent patterns. Python libraries such as GUDHI and Ripser were used for TDA analysis [19]. Both the 0th and 1st dimensions were analyzed, focusing on the connected components (H0) and loops (H1). The filtration range was adjusted according to the questionnaire response data to capture the significant topological features. The analysis revealed multi-loop structures, suggesting multiple pathways for NYT's therapeutic effects [20].

Network graph clustering

Following the TDA, the relationships between different health-related questions were further explored using network graph clustering. This process allowed us to visualize how patient responses across multiple health domains were interconnected. To represent the relationships between the questions, we used Python libraries designed for the construction and manipulation of complex networks. In this graph, each question was represented as a node, and edges between nodes indicated the strength of correlations between the responses. Additionally, eigenvector centrality was calculated to evaluate the influence of each question within the network. This method assesses a node's importance based on the importance of its neighboring nodes, allowing us to identify the most influential questions in the network. To detect clusters within this network, we applied the Louvain method for community detection. This algorithm identifies groups of highly interconnected nodes, representing distinct "communities" of related health effects. The Louvain method optimizes the modularity score, which indicates how well the network is divided into communities. A higher modularity score suggests stronger intra-community connections compared to inter-community connections. The methodology and code for this analysis are publicly available for further validation and use [21].

Statistical analysis

The primary statistical analysis was designed to assess the effect of NYT on multiple health domains in patients with frailty. Descriptive statistics were used to summarize demographic characteristics, baseline clinical data, and questionnaire responses. Categorical variables are presented as frequencies and percentages, while continuous variables are expressed as means with standard deviations (SD). Paired t-tests were applied to compare pre- and post-treatment questionnaire scores to assess significant differences before and after NYT administration. To account for multiple comparisons, a Bonferroni correction was applied, adjusting the significance threshold to 0.001786 (0.05/28). The detailed methodology and code for TDA and network graph clustering, which were used to explore the relationships between health outcomes, are provided in the Appendix. All statistical analyses were performed using Python, and the relevant code was publicly available for further validation and use.

## Results

Patients

An overview of the 15 patients, including their key demographic and clinical characteristics, is presented below. The average age of the participants was 82.67 ± 4.27 years, and their average BMI was 21.73 ± 2.55 kg/m². Among the 15 participants, 14 (93.3%) had hypertension, and five (33.3%) had diabetes. No cases of severe renal impairment or liver dysfunction were reported among the participants.

Table 2 shows the average and SD of laboratory parameters for the participants. The results show that while most patients had well-maintained hematological and biochemical parameters, some patients had slightly elevated HbA1c and triglyceride levels.

**Table 2:** Average and SD of laboratory parameters for participants. WBC: white blood cells; RBC: red blood cells; Hb: hemoglobin; Ht: hematocrit; MCV: mean corpuscular volume; MCH: mean corpuscular hemoglobin; MCHC: mean corpuscular hemoglobin concentration; APTT: activated partial thromboplastin time; FDP: fibrin degradation products; TP: total protein; AST: aspartate aminotransferase; ALT: alanine aminotransferase; LD: lactate dehydrogenase; ALP: alkaline phosphatase; γ-GT: gamma-glutamyl transferase; Cr: creatinine; UA: uric acid; UN: urea nitrogen; HbA1c: hemoglobin A1c; TG: triglycerides; HDL: high-density lipoprotein; SD: standard deviation. All values in the table are expressed as mean ± SD, providing a comprehensive overview of each parameter's average and variability across participants.

Parameter with units	Reference range	Mean±SD
WBC (×10³/µL)	3500-9700	5861.11 ± 799.78
RBC (×10⁶/µL)	376-516	415.67 ± 42.8
Hb (g/dL)	11.2-15.2	12.44 ± 1.19
Ht (%)	34.3-45.2	39.79 ± 3.56
Platelets (×10³/µL)	14.0-37.9	25.14 ± 5.39
MCV (fL)	80-101	95.89 ± 2.96
MCH (pg)	26.4-34.3	30.0 ± 1.14
MCHC (g/dL)	31.3-36.1	31.28 ± 0.49
APTT (sec)	26.0-38.0	30.89 ± 3.34
Fibrinogen (mg/dL)	170-410	323.83 ± 46.41
FDP (µg/mL)	0-5	3.14 ± 1.12
TP (g/dL)	6.5-8.2	7.01 ± 0.49
AST (U/L)	10-40	25.0 ± 4.19
ALT (U/L)	5-45	15.56 ± 2.95
LD (U/L)	120-245	217.11 ± 33.38
ALP (U/L)	38-113	79.33 ± 13.94
γ-GT (U/L)	0-48	33.33 ± 26.93
Cr (mg/dL)	0.46-0.82	0.65 ± 0.09
UA (mg/dL)	2.7-7.0	4.71 ± 1.14
UN (mg/dL)	8.0-20.0	20.58 ± 3.83
HbA1c (%)	4.6-6.2	6.14 ± 0.71
TG (mg/dL)	50-149	109.56 ± 33.31
Total cholesterol (mg/dL)	150-219	195.56 ± 32.54
HDL cholesterol (mg/dL)	40-90	59.67 ± 12.09
Testosterone (ng/dL)	10.8-56.9	14.67 ± 1.45

Improvement points by question

To understand the individual variability in the effects of NYT among patients with frailty and OAB, we first analyzed the distribution of improvement points across the 28 questions included in the simplified questionnaire. Each question reflects a specific domain of health, and patients were asked to identify the effects they experienced after NYT administration. The results are summarized in Table 3, which shows the number and percentage of patients who reported improvement for each question.

**Table 3:** Distribution of improvement points across questions in patients with frailty and OAB after NYT administration. NYT: Ninjin'yoeito; OAB: overactive bladder. Question no.: Refers to the specific question number in the simplified questionnaire, each targeting a specific health domain. Number of patients with effect (%): Represents the number of patients who reported experiencing an improvement for that particular question, with the percentage indicating the proportion of the total patient population. Improvement points: Indicates areas where patients reported feeling better or experiencing beneficial effects from NYT administration.

Question no.	Number of patients with effect (%)
1	13 (86%)
2	10 (66%)
3	11 (73%)
4	4 (26%)
5	2 (13%)
6	0 (0%)
7	0 (0%)
8	1 (7%)
9	5 (33%)
10	6 (40%)
11	8 (53%)
12	8 (53%)
13	8 (53%)
14	7 (46%)
15	5 (33%)
14	7 (46%)
15	5 (33%)
16	6 (40%)
17	2 (13%)
18	0 (0%)
19	0 (0%)
20	0 (0%)
21	2 (13%)
22	2 (13%)
23	1 (7%)
24	0 (0%)
25	11 (73%)
26	10 (66%)
27	13 (86%)
28	12 (80%)

Significant improvements after NYT administration 

To further substantiate these findings, a paired t-test was conducted across all 28 questions to assess the statistical significance of the differences between pre- and post-treatment responses. To account for multiple comparisons and reduce the risk of type I errors, a Bonferroni correction was applied, adjusting the significance level (alpha) accordingly. The corrected significance threshold was set to 0.001786 (0.05/28 tests). Statistically significant improvements were observed in several key areas even after Bonferroni correction. For example, questions related to energy and vitality, including the frequency of feeling fatigued in daily life (Question 1, p = 0.000002), increased vitality (Question 2, p = 0.000291), and energy levels during physical activity (Question 3, p = 0.000075), showed notable improvements after NYT administration. Similarly, improvements in muscle strength and physical function were evident, as questions related to changes in muscle strength (Question 9, p = 0.000010), daily physical strength (Question 10, p = 0.000012), and stamina when climbing stairs (Question 12, p = 0.000031) demonstrated statistically significant differences. Mental health indicators also showed positive changes, with significant improvements in the frequency of mental stress (Question 13, p = 0.000002), feelings of depression (Question 14, p = 0.000003), and anxiety levels (Question 15, p = 0.000001). Additionally, all questions related to OAB, including daytime urination frequency (Question 25, p = 0.000002), nighttime urination frequency (Question 26, p = 0.000075), and experiences of urgency and incontinence (Questions 27 and 28, p = 0.000001 and p = 0.000005, respectively), demonstrated statistically significant improvements. Conversely, no significant difference was found in the question regarding reductions in catching colds or infections (Question 5, p = 0.164318), as the p-value exceeded the Bonferroni-corrected threshold.

Individual improvement selections

All patients were asked to identify approximately three questions from the 28-item questionnaire in which they felt the strongest effects of the NYT. This personalized selection of improvement points is crucial for subsequent topological analysis, as it directly reflects individual variability in response to NYT. By analyzing these selections, we aimed to map the relationships between the questions and visualize the diverse effects of NYT across different health domains. This approach not only highlights the specific areas where NYT has the most impact but also underscores the variation in patient experiences, providing a more detailed and individualized view of NYT's therapeutic effects. Table 4 shows the individual improvement selections made by each patient, indicating the questions where they felt the most improvement from NYT administration.

**Table 4:** Individual improvement selections in response to NYT among patients with frailty. NYT: Ninjin'yoeito. Patient: Each row represents a different patient in the study. Selected questions: Lists the specific questions chosen by each patient where they felt the most improvement from NYT. These selections indicate the areas of greatest perceived benefit and are used for further analysis of individual responses.

Patient	Selected questions
Patient 1	Question 1, Question 6, Question 15, Question 27
Patient 2	Question 9, Question 12, Question 26
Patient 3	Question 8, Question 21, Question 22, Question 26
Patient 4	Question 2, Question 11, Question 27
Patient 5	Question 3, Question 17, Question 28
Patient 6	Question 1, Question 23
Patient 7	Question 2, Question 11, Question 25
Patient 8	Question 2, Question 15, Question 27
Patient 9	Question 1, Question 4, Question 12, Question 27
Patient 10	Question 1, Question 14, Question 28
Patient 11	Question 3, Question 12, Question 25
Patient 12	Question 9, Question 12, Question 13, Question 25
Patient 13	Question 1, Question 15, Question 17
Patient 14	Question 4, Question 10, Question 27
Patient 15	Question 1, Question 13, Question 25

TDA persistence diagram

**Figure 1:**
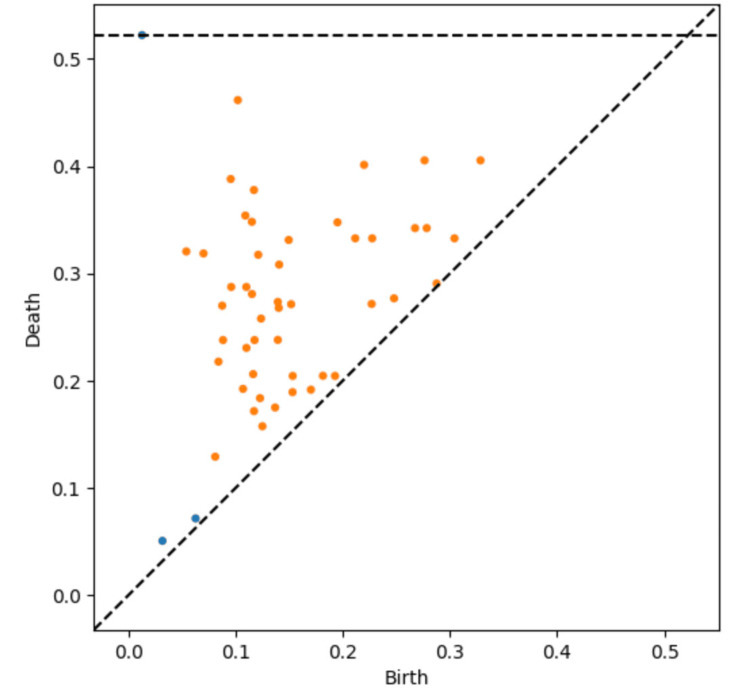
Persistence diagram of topological features from TDA analysis. This figure illustrates the "birth" and "death" of topological features identified through TDA using persistent homology. It indicates multiple interconnected pathways of improvement across different health domains. X-axis: birth scale, where features appear. Y-axis: death scale, where features disappear. Dashed diagonal line indicates where birth equals death, highlighting features with minimal persistence. Clusters (H₀) are represented as blue points, while loops or cycles (H₁) are shown as orange points. Prevalence of H₁ over H₀ suggests that NYT's therapeutic effects manifest through complex, multi-loop structures. TDA: topological data analysis.

To further explore the individual variability and complex effects of NYT, we performed a TDA using persistent homology. TDA allowed us to investigate the interconnectedness and persistence of the NYT’s effects across different health domains. The persistent homology analysis revealed a complex, multi-loop structure in the data, suggesting multiple pathways through which NYT exerts its therapeutic benefits. Specifically, the persistence diagram (Figure 1) shows the "birth" and "death" of topological features such as clusters (H₀) and loops (H₁). The analysis highlighted a significant prevalence of H₁ features, corresponding to loops or cycles, over H₀ features, which represent clusters. This indicates that NYT’s therapeutic effects manifest in a multi-loop structure, reflecting the diverse and interconnected nature of its impact on various health domains. The abundance of these loops emphasizes the multifaceted nature of NYT’s effects, demonstrating that the benefits are not limited to simple, isolated improvements but are interconnected across physical, mental, and functional health domains. This complexity underscores the need for personalized medical approaches when administering NYT, as different patients may experience improvement through various overlapping pathways. Figure 1 shows the persistence diagram generated from the analysis, which visually captures the intricate relationships and persistence of NYT’s effects over time.

Network graph clustering 

Following the TDA persistence diagram, a network graph was constructed to visualize the relationships between different health-related questions based on patient responses. This graph highlights the connections between the questions and reveals the distinct communities of related health domains. Using Python libraries, such as NetworkX for graph construction and the Louvain method for community detection, we identified specific clusters exhibiting strong correlations. Each question is represented as a node, and the edges between them denote the strength of their connections.

To assess the importance of individual questions within the network, eigenvector centrality was calculated. Question 1 showed the highest eigenvector centrality (0.444963), indicating that it has the strongest influence in the network. Question 12 also demonstrated significant importance with an eigenvector centrality score of 0.371986, suggesting it plays a key role in connecting various health domains. Additionally, betweenness centrality was calculated to identify questions that serve as bridges between different parts of the network. Question 1 once again emerged as highly central, with a betweenness centrality score of 0.350702, while Question 12 had a score of 0.349737, further confirming its critical role.

**Figure 2:**
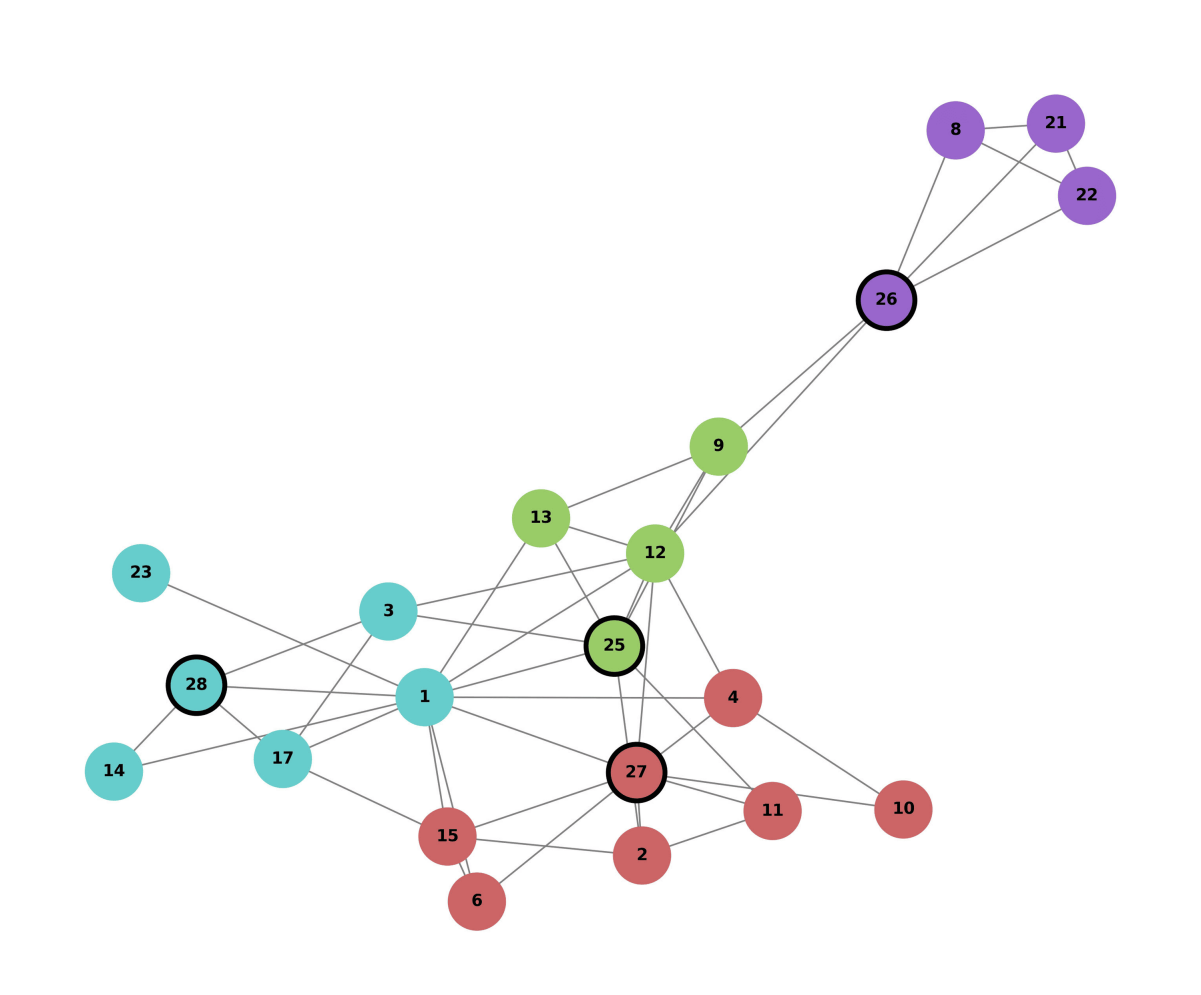
Topological network graph of health-related questions and community structure. 1-4: Energy and Vitality Improvement (SF-36, Fatigue Assessment Scale); 5-8: Immune Function Improvement (Self-Rated Health Questionnaire); 9-12: Muscle Strength and Physical Function Improvement (Frailty Evaluation Scale); 13-16: Mental Stress Reduction (DASS-21, GDS); 17-20: Digestive Function Improvement (GSRS); 21-24: Sleep Quality Improvement (PSQI); 25-28: Improvement of Overactive Bladder Symptoms (OABSS). The nodes are colored according to their community affiliation, identified using the Louvain method for community detection. Specific nodes related to OABSS (Questions 25, 26, 27, and 28) are highlighted to emphasize their significance in the analysis. Edges between nodes represent the strength of connections based on correlations derived from patient responses, indicating the relationships between different health domains. SF-36: Short Form-36; DASS-21: Depression Anxiety Stress Scales-21; GDS: Geriatric Depression Scale; GSRS: Gastrointestinal Symptom Rating Scale; PSQI: Pittsburgh Sleep Quality Index; OABSS: Overactive Bladder Symptom Score.

The modularity score for this clustering was 0.387, indicating that the identified communities are well separated and the clustering was effective. Figure 2 shows the resulting network graph, where questions related to the OABSS were particularly emphasized, including questions 25, 26, 27, and 28, which were outlined with thick black borders to highlight their significance in the analysis. The community detection analysis revealed four distinct clusters of interconnected health domains.

The first cluster was primarily associated with energy and vitality improvement as well as muscle strength and physical function improvement. This group included questions related to daily fatigue, energy levels during physical activity, and muscle strength changes, along with questions related to OAB symptoms. This finding suggests a strong link between physical vitality and urinary health.

The second cluster encompasses a broader range of health aspects, including immune function improvement, digestive function improvement, sleep quality improvement, and mental stress reduction. This group indicated a holistic approach to health improvement, highlighting the wide-ranging effects of NYT on both physical and mental well-being.

The third cluster focused on muscle strength and physical function improvement in conjunction with mental stress reduction. It includes questions about muscle strength, physical function, and anxiety levels. The presence of OAB-related questions in this group underscores the complex interplay between the physical and psychological factors in managing these symptoms.

The final cluster was dominated by questions related to sleep quality improvement, particularly covering aspects such as sleep quality, nighttime awakening, and morning fatigue. The inclusion of OAB-related questions in this cluster highlights the impact of urinary symptoms on sleep and illustrates the interconnectedness of these issues.

This network analysis underscores the diverse and overlapping effects of NYT on multiple health domains. This finding demonstrates the importance of addressing various interconnected health aspects to provide more comprehensive care. By mapping the relationships between health domains, this analysis offers valuable insights into the complex interactions between NYT effects and how they can vary across individuals. This approach highlights the need for personalized and effective treatment strategies.

## Discussion

This pilot study introduces a novel approach to clinical medical research, particularly for evaluating complex Kampo medicines, such as NYT. Combining TDA with conventional methods elucidated intricate patterns of NYT's effects on patients with frailty and OAB symptoms, offering new insights into personalized medicine and the holistic nature of Kampo medicine [1,3]. The application of TDA in clinical medicine represents a significant methodological advancement [12,18]. TDA addresses many constraints of traditional statistical approaches by capturing complex interrelationships, handling nonlinear data, and integrating multidimensional datasets [12,22]. This method is particularly effective in studying Kampo medicines such as NYT, where the effects are multifaceted and prone to individualization [3,4]. Furthermore, recent literature reports specific clinical cases demonstrating NYT's therapeutic effects in elderly female patients with frailty and OAB, showing improvements in physical function, cognitive function, and sleep quality [9].

Our innovative approach of using NLP to simplify multiple standardized questionnaires into a 28-item format provides a practical solution to common challenges in clinical research involving patients with frailty [10]. This method allows for comprehensive data collection while reducing patient burden, enabling a more holistic assessment of the effects of NYT [1,4]. Persistent homology analysis revealed complex multi-loop structures, indicating diverse pathways of NYT's effects [13,19]. The prevalence of H1 (loops) over H0 (clusters) features suggests that NYT's effects are interconnected across different health domains rather than isolated improvements in specific symptoms [20,23-25]. This aligns with the holistic philosophy of Kampo medicine and provides a mathematical foundation for understanding its complex mechanisms of action [3].

Network graph clustering analysis using the Louvain method enables the visualization of relationships between different health domains influenced by NYT [11,12]. The four distinct communities that emerged in the graph demonstrate the interconnectedness of NYT's effects, reminiscent of community structures observed in famous social network models, such as Zachary's karate club [21,26,27]. One community linked energy, vitality, and OAB symptoms, suggesting a potential relationship between physical function and urinary health [1,8]. Another community has highlighted NYT's comprehensive effects on both physical and mental health [4,5]. The third community pointed to the interrelations between physical function, mental stress, and OAB symptoms, while the fourth emphasized the impact of urinary health on sleep quality [1,14]. This visualization supports the holistic approach of Kampo medicine and provides a framework for understanding the complex interactions between different health domains in individual patients [3,4,21,28].

Our approach also revealed the limitations of summarizing NYT effects with mean ± SD, which might have suggested uniform effects across all domains. By focusing on the number of patients reporting improvement for each question, we clearly demonstrated the diversity of NYT effects [1,7]. This underscores the importance of methods that capture individual variations, especially in complex interventions, such as Kampo medicines [3,6].

This study presents a novel method to mathematically express the holistic approach of Kampo medicine [3,13,19]. By visualizing the interrelations between multiple health domains and individual response patterns, we can bridge Eastern and Western medicine, potentially deepening our understanding of complex medical interventions [3,4]. Combining TDA with traditional clinical research methods represents a new paradigm in Kampo medicine [13,19], providing a rigorous and quantitative evaluation framework that aligns with its philosophical foundations [13,19]. These methods may pave the way for more sophisticated and individualized approaches in Kampo medicine research and applications [3,4].

These findings have significant implications for personalized medicine. The ability to visualize and analyze individual response patterns to NYT could lead to individualized treatment strategies, potentially enhancing the efficacy and reducing side effects [1,4,8]. Moreover, this approach could lead to the development of predictive models for treatment responses, which is a key goal in personalized medicine [10,13,29,30].

This pilot study exhibited several limitations. As an initial investigation, our findings necessitate validation through large-scale studies involving diverse patient populations. The brief duration of NYT administration may not adequately capture long-term effects or adverse reactions. Moreover, the absence of a control group hindered the attribution of observed effects solely to NYT, as placebo effects or the natural progression of the disease may have influenced the results. It is imperative to investigate whether the simplified questionnaire yields comparable results to the original questionnaire. TDA is a powerful analytical tool that provides novel insights into complex interrelationships, and its application holds promise for future research. In this study, we evaluated the effectiveness of TDA through a small-scale pre-post comparison. However, patients with frailty often have unstable health conditions, necessitating family members to take time off work for each visit, thus making randomized controlled trials (RCTs) challenging to conduct. Future research should incorporate strategies such as home visits, enabling large-scale RCTs to rigorously validate the effects of NYT.

## Conclusions

This pilot study demonstrated the feasibility of using TDA and NLP to analyze the complex effects of NYT on frailty in patients with an OAB. Our findings revealed multi-loop structures in the therapeutic effects of NYT, indicating multiple pathways of improvement across physical and mental health domains. This novel approach provides a more comprehensive understanding of the holistic nature of Kampo medicine and offers new insights into personalized treatment strategies for frailty and OAB. Future large-scale studies employing these advanced analytical methods could further elucidate the long-term efficacy of NYT and potentially revolutionize the evaluation and application of complex interventions in clinical medicine.

## Appendices

Comparison between classical statistical methods and new analytical approaches

To provide a more comprehensive understanding of the analytical methods used in this study, we have included this appendix. While the code used for NLP, network graph analysis, and TDA is publicly available, it is essential to explain why these modern techniques were chosen and how they differ from traditional statistical approaches. This comparison helps clarify the strengths of each method and provides context for the complexity of the effects of NYT on frailty in patients with overactive bladder (OAB). By presenting this detailed comparison, we aim to enhance the clarity and reproducibility of the study, ensuring a better understanding of the rationale behind our methodological choices.

1. Classical Statistical Methods

Classical statistical methods, such as linear regression and hypothesis testing, are widely used to model relationships between variables. They are particularly effective for identifying clear patterns or differences between groups when the data structure is relatively simple. For example, a t-test is commonly applied to determine whether the means of two groups are significantly different. The formula for the t-test is as follows:

$t = \frac{\bar{X}_1 - \bar{X}_2}{\sqrt{\frac{s_1^2}{n_1} + \frac{s_2^2}{n_2}}}$

Where *X*_1_*,X*_2_ are the means of two groups, *s*_1_,*s*_2_​ are the standard deviations, and *n*_1_,*n*_2_*​* are the sample sizes of the two groups.

Linear regression is another common method, where the relationship between the dependent variable *y* and independent variable *x* is expressed as follows:

$y = \beta_0 + \beta_1 x + \epsilon$

Where *y* is the dependent variable, *x* is the independent variable, *β_0_*​ is the intercept, *β_1_​* is the slope of the regression line, and *ϵ* is the error term.

These methods are useful for identifying specific patterns or differences between groups but have limitations when analyzing complex multidimensional structures in data. Classical statistical methods, such as t-tests and linear regression, are valuable for testing specific hypotheses but are limited when it comes to analyzing complex, high-dimensional data. In our study, traditional statistical tools were used, such as paired t-tests to compare pre- and post-treatment responses, and the Bonferroni correction to adjust for multiple comparisons, with a significance threshold of 0.001786. While these approaches showed clear differences in specific questionnaire responses, they are inherently linear and are not designed to handle the nonlinear, multidimensional nature of the data we collected. The interactions between different health domains, such as mental stress and physical strength, do not lend themselves to straightforward linear models.

For example, a t-test can only tell us whether the average response to a particular question significantly changed after treatment. However, it does not reveal how different health domains interact, such as how improvements in muscle strength might correlate with better sleep quality or reduced anxiety. Classical methods struggle to model these complex, networked relationships.

2. Natural Language Processing

In this study, natural language processing (NLP) was used to simplify and condense a set of extensive health questionnaires. Classical statistical methods do not provide a straightforward way to handle such high-dimensional, textual data. To address this, we applied term frequency-inverse document frequency (TF-IDF) to evaluate the relative importance of each question. The formula for TF-IDF is as follows:

$\text{TF-IDF}(t,d) = \text{TF}(t,d) \times \log \left( \frac{N}{\text{DF}(t)} \right)$

Where *TF*(*t, d*) is the term frequency of term *t* in document *d*, *N* is the total number of documents, *DF*(*t*) is the number of documents in which term *t* appears.

To reduce dimensionality and group questions into themes, singular value decomposition (SVD) was applied. SVD is expressed as follows:

$X = U \Sigma V^{T}$

Where *X* is the original TF-IDF matrix, *U* and V^T^ are orthogonal matrices, and *Σ* is the diagonal matrix of singular values.

This method condensed the original set of 135 questions into four main themes, making the questionnaire manageable while maintaining its evaluative power.

3. Network Graph Analysis

Network graph analysis was used to explore the complex relationships between different health domains based on patient responses. In this study, each health-related question was treated as a node, and the correlations between questions were represented by edges between the nodes. To detect communities, we applied the Louvain method, which optimizes modularity-a measure of the strength of division of a network into communities. The modularity Q is calculated as follows:

$Q = \frac{1}{2m} \sum_{ij} \left[ A_{ij} - \frac{k_i k_j}{2m} \right] \delta(c_i, c_j)$

Where *Q* is the modularity score, *A_ij_* is the weight of the edge between nodes *i* and *j*, *k_i_* and *k_j​_* are the degrees of nodes *i* and *j*, *m* is the total number of edges in the graph, and *δ(c_i_,c_j_)* is 1 if nodes *i* and *j* are in the same community and 0 otherwise.

Additionally, eigenvector centrality measures the influence of a node based on the influence of its neighbors: $C(v_i) = \frac{1}{\lambda} \sum_{v_j \in N(v_i)} A_{ij} C(v_j)$

Where *λ* is the eigenvalue, *A_ij_* is the adjacency matrix, and *C*(*v_j_*) is the centrality of node *v_j​_*.

Betweenness Centrality measures how often a node lies on the shortest path between two other nodes:

$BC(v) = \sum_{s \neq v \neq t} \frac{\sigma_{st}(v)}{\sigma_{st}}$

Where *σ_st_(v)* is the number of shortest paths between *s* and *t* that pass through *v*, and *σ_st_*​ is the total number of shortest paths between *s* and *t*.

In our case, this method identified four distinct clusters of related health domains, with a modularity score of 0.387. This means that the clustering was effective, with each cluster representing a community of questions that shared stronger connections internally than with other questions. Additionally, eigenvector centrality was calculated to identify the most influential questions within the network. Question 1 emerged as the most central question, with the highest eigenvector centrality (0.444963), indicating its strong influence across multiple health domains. Question 12 also showed significant importance with an eigenvector centrality of 0.371986. Betweenness centrality further revealed that Question 1 and Question 12 played key roles as bridges connecting different clusters, with betweenness centrality scores of 0.350702 and 0.349737, respectively. This approach allowed us to visualize how the effects of NYT were distributed across energy, physical function, mental stress, and sleep quality-insights that traditional statistical methods would have missed.

4. Topological Data Analysis

Topological data analysis (TDA) provides a unique approach to understanding the shape and structure of high-dimensional data. In this study, persistent homology was applied to analyze the topological features of patient health data, capturing both local and global structures that remain persistent across multiple scales. The key concept in TDA is the Betti number, which represents the number of topological features (such as clusters or loops) in different dimensions. The Betti number β_k_​ is calculated as follows:

$\beta_k = \text{rank}(\ker \partial_k) - \text{rank}(\text{im} \, \partial_{k+1})$

Where *β_k_*​ represents the number of k-dimensional holes (such as loops or clusters) and *∂_k_*​ is the boundary operator for dimension *k*.

The results of persistent homology are visualized using persistence diagrams, which plot the birth and death of topological features, showing when a cluster or loop forms and disappears. The persistence diagram produced by TDA showed a multiloop structure in the effects of NYT, indicating multiple pathways of improvement that were interlinked. This structure provides a holistic view of how NYT influenced physical, mental, and functional health domains in a way that traditional linear models simply cannot capture. The persistence of these features across multiple scales suggests that NYT's effects are not isolated but part of a broader, interconnected therapeutic pattern.

5. Why Classical Methods Alone Fall Short

While classical statistical methods are valuable for testing specific hypotheses, they are inherently limited in their ability to analyze complex, interconnected datasets like those generated in our study. The use of t-tests and regression provided important insights into specific pre- and post-treatment differences, but they lacked the ability to model nonlinear interactions between different health domains. Although the Bonferroni correction was applied to reduce type I errors, it further emphasizes the reductionist nature of classical methods, which focus on independent significance testing rather than the interconnectedness of variables. In contrast, modern methods such as NLP, network graph analysis, and TDA allowed us to uncover multidimensional relationships and persistent patterns in the data, providing a richer and more nuanced understanding of NYT's effects. These methods align more closely with the holistic philosophy of Kampo medicine, which considers the interdependence of physical, mental, and functional health.

By combining these modern analytical techniques, we gained a much deeper understanding of the complex effects of NYT on patients with frailty and OAB. While classical statistical methods were useful in identifying individual differences, they could not reveal the multiloop, multidimensional structures uncovered by TDA or the community-based relationships highlighted through network graph analysis. This appendix demonstrates the value of applying advanced data science methods to clinical research, especially when studying interventions like NYT, which have broad, individualized effects. By using NLP, network analysis, and TDA, we were able to provide a holistic view of NYT’s therapeutic effects, offering new insights for personalized medicine. These approaches will be critical in future studies that aim to capture the complex interactions in similar datasets, and they provide a robust alternative to classical methods for understanding complex medical interventions.

## References

[1] Okui N, Okui MA (2023). Ninjin'yoeito improves genitourinary symptoms in patients with frailty. Cureus.

[2] Fukuda E, Misugi T, Kitada K (2022). The hematopoietic effect of Ninjinyoeito (TJ-108), a traditional Japanese herbal medicine, in pregnant women preparing for autologous blood storage. Medicina (Kaunas).

[3] Takayama S, Arita R, Ohsawa M (2019). Perspectives on the use of Ninjin'yoeito in modern medicine: a review of randomized controlled trials. Evid Based Complement Alternat Med.

[4] Okahara K, Ohsawa M, Haruta-Tsukamoto A (2023). Frailty improvement by multicomponent drug, Ninjin'Yoeito, in mild cognitive impairment and mild Alzheimer's disease patients: an open-label exploratory study (FRAMINGO). J Alzheimers Dis Rep.

[5] Yagi T, Sawada K, Miyamoto M (2022). Safety and efficacy of Ninjin'yoeito along with iron supplementation therapy for preoperative anemia, fatigue, and anxiety in patients with gynecological disease: an open-label, single-center, randomized phase-II trial. BMC Womens Health.

[6] Nakagawa Y, Kagohashi K, Shioya A, Kinoshita K, Tsuji H, Satoh H (2022). Maintaining weight and nutritional status with Ninjin'yoeito in elderly patients with chronic wasting diseases. Sultan Qaboos Univ Med J.

[7] Sakisaka N (2022). Long-term administration of Ninjin'yoeito to treat frailty in older adults: a case series. Neuropeptides.

[8] Ohbayashi H, Ariga M, Ohta K, Kudo S, Furuta O, Yamamoto A (2024). Effects of Ninjin'yoeito on patients with chronic obstructive pulmonary disease and comorbid frailty and sarcopenia: a preliminary open-label randomized controlled trial. Int J Chron Obstruct Pulmon Dis.

[9] Okui N (2024). Ninjin'yoeito in the management of frailty and overactive bladder in elderly women: a report of two cases. Cureus.

[10] van Buchem MM, Neve OM, Kant IM, Steyerberg EW, Boosman H, Hensen EF (2022). Analyzing patient experiences using natural language processing: development and validation of the artificial intelligence patient reported experience measure (AI-PREM). BMC Med Inform Decis Mak.

[11] Blondel VD, Guillaume EL, Lambiotte R, Lefebvre E (2008). Fast unfolding of communities in large networks. J Stat Mech Theor Exp.

[12] Fortunato S (2010). Community detection in graphs. Physics Rep.

[13] Ravishanker N., Chen R (2021). An introduction to persistent homology for time series. Wiley Interdiscip Rev Comput Stat.

[14] Mollayeva T, Thurairajah P, Burton K, Mollayeva S, Shapiro CM, Colantonio A (2016). The Pittsburgh Sleep Quality Index as a screening tool for sleep dysfunction in clinical and non-clinical samples: a systematic review and meta-analysis. Sleep Med Rev.

[15] Op Het Veld LP, de Vet HC, van Rossum E, Kempen GI, van Kuijk SM, Beurskens AJ (2018). Substitution of Fried's performance-based physical frailty criteria with self-report questions. Arch Gerontol Geriatr.

[16] Buck E (2014). Critical synthesis package: Depression Anxiety Stress Scales (DASS). MedEdPORTAL.

[17] Wancata J, Alexandrowicz R, Marquart B, Weiss M, Friedrich F (2006). The criterion validity of the Geriatric Depression Scale: a systematic review. Acta Psychiatr Scand.

[18] Parijs B, Price M, León F, Fehnel S (2008). Relevance of the Gastrointestinal Symptom Rating Scale (GSRS) in patients with celiac disease 1009. Am J Gastroenterol.

[19] (2024). Okuinobuo: NYT20240901. https://github.com/Okuinobuo/NYT20240901/.

[20] Cohen-Steiner D, Edelsbrunner H, Harer J (2005). Stability of persistence diagrams. Discrete Comput Geom.

[21] Akama H, Miyake M, Jung J, Brian M: Friendship network of Zachary’s famous Karate Club. PLOS ONE (2015). Akama H, Miyake M, Jung J, Brian M: Friendship network of Zachary’s famous Karate Club. PLOS ONE. http://10.1371/JOURNAL.PONE.0125725.G001.

[22] Tsaneva-Atanasova K, Scotton C (2023). How to handle big data for disease stratification in respiratory medicine?. Thorax.

[23] Bhaskar D, Zhang WY, Wong IY (2021). Topological data analysis of collective and individual epithelial cells using persistent homology of loops. Soft Matter.

[24] Bhaskar D, Zhang WY, Volkening A, Sandstede B, Wong IY (2023). Topological data analysis of spatial patterning in heterogeneous cell populations: clustering and sorting with varying cell-cell adhesion. NPJ Syst Biol Appl.

[25] El-Yaagoubi AB, Chung MK, Ombao H (2023). Topological data analysis for multivariate time series data. Entropy (Basel).

[26] Al-Mukhtar AF, Al-Shamery ES (2018). Greedy modularity graph clustering for community detection of large co-authorship network. Int J Eng Technol.

[27] Kramer J, Boone L, Clifford T, Bruce J, Matta J (2020). Analysis of medical data using community detection on inferred networks. IEEE J Biomed Health Inform.

[28] Matta J, Singh V, Auten T, Sanjel P (2023). Inferred networks, machine learning, and health data. PLoS One.

[29] Bukkuri A, Andor N, Darcy IK (2021). Applications of topological data analysis in oncology. Front Artif Intell.

[30] Okui N (2024). Innovative decision making tools using discrete mathematics for stress urinary incontinence treatment. Sci Rep.

